# Development of a surgical procedure for removal of a placentome from a pregnant ewe during gestation

**DOI:** 10.1186/s40104-020-00454-1

**Published:** 2020-05-14

**Authors:** Colleen A. Lambo, Ashley K. Edwards, Fuller W. Bazer, Kathrin Dunlap, M. Carey Satterfield

**Affiliations:** 1 Veterinary Physiology and Pharmacology, College of Veterinary Medicine and Biomedical Sciences Texas A&M University, College Station, TX 77843 USA; 2 Department of Animal Science, Texas A&M University, 2471 TAMU, College Station, TX 77843 USA

**Keywords:** Developmental biology, Fetal development, IUGR, Ovine/sheep, Placenta, Placental transport

## Abstract

**Background:**

In recent decades, there has been a growing interest in the impact of insults during pregnancy on postnatal health and disease. It is known that changes in placental development can impact fetal growth and subsequent susceptibility to adult onset diseases; however, a method to collect sufficient placental tissues for both histological and gene expression analyses during gestation without compromising the pregnancy has not been described. The ewe is an established biomedical model for the study of fetal development. Due to its cotyledonary placental type, the sheep has potential for surgical removal of materno-fetal exchange tissues, i.e., placentomes. A novel surgical procedure was developed in well-fed control ewes to excise a single placentome at mid-gestation.

**Results:**

A follow-up study was performed in a cohort of nutrient-restricted ewes to investigate rapid placental changes in response to undernutrition. The surgery averaged 19 min, and there were no viability differences between control and sham ewes. Nutrient restricted fetuses were smaller than controls (4.7 ± 0.1 kg vs. 5.6 ± 0.2 kg; *P* < 0.05), with greater dam weight loss (− 32.4 ± 1.3 kg vs. 14.2 ± 2.2 kg; *P* < 0.01), and smaller placentomes at necropsy (5.7 ± 0.3 g vs. 7.2 ± 0.9 g; *P* < 0.05). Weight of sampled placentomes and placentome numbers did not differ.

**Conclusions:**

With this technique, gestational studies in the sheep model will provide insight into the onset and complexity of changes in gene expression in placentomes resulting from undernutrition (as described in our study), overnutrition, alcohol or substance abuse, and environmental or disease factors of relevance and concern regarding the reproductive health and developmental origins of health and disease in humans and in animals.

## Introduction

For the past half century, researchers have performed terminal procedures on ruminants to investigate placentome development and its impacts on fetal growth and health [1]. More recent studies have delved into gene expression in placentomes in early stages of gestation with similar goals [2, 3]. Acquisition of information from early and pivotal stages of placental development will advance our understanding of factors that are required for or are detrimental to optimal fetal growth and maturation. The draw-back for any terminal procedures, however, is an inability to correlate placentome growth in early- to mid-gestation with fetal development at term, or, optimally, to morbidity, mortality, and efficiency of growth and development of the resulting offspring. Non-terminal investigations have sought to evaluate parameters of placental and placentome growth and development using Doppler blood flow methods [4, 5], but placentome sampling during gestation without compromising fetal-placental development has not been reported. For the purposes of this communication, placentome will refer to entire discrete regions of interdigitating fetal (cotyledon) and maternal (caruncle) tissues [6].

The ewe has been hailed as a valuable research model for studies relevant to human pregnancy [7, 8]; therefore, knowledge of placental/fetal interactions and how treatments or diseases impact them is critical for understanding, diagnosing, and preventing, causes and effects of deleterious events in placental development which occurs during early- to mid-gestation. Medical concerns regarding intrauterine growth restriction (IUGR), fetal alcohol syndrome, environmental toxins and pollutants, and other conditions associated with compromised pregnancy are lifelong medical issues that need to be assessed and understood from the earliest stages of gestational programming. Many of the described experimental insults produce spectral phenotypes, which can perhaps best be understood by retrospectively comparing the phenotype to the conditions that existed during early stages of fetal-placental development and before the phenotype of the live offspring is established. A non-terminal surgical technique that allows for sampling of placentomes from a pregnant ewe would provide critical knowledge related to gene-environmental interactions affecting fetal-placental development during gestation and how those interactions impact lifelong health of that offspring.

One area in which research with ewes has proven to be of benefit for human healthcare concerns is that of IUGR, or low birth-weight infants. Our laboratory has developed a nutrient-restricted sheep model for studying IUGR risks in humans [9, 10]. As a proof of concept, initial trials with this novel surgical procedure were performed with ewes in which either a placentome was removed (placentomectomy) or a sham-surgery was performed. A second study tested the efficacy of the surgical approach in our established nutrient-restricted ewe model.

## Materials & methods

### Animals and experimental protocols

All experimental and surgical procedures were in compliance with the Guide for the Care and Use of Agriculture Animals in Research and Teaching and approved by the Institutional Animal Care and Use Committee of Texas A&M University.

Embryos were generated from superovulated Hampshire ewes (*n* = 37), of similar body weight and frame size, utilizing semen from four half-sibling rams [11]. Single grade-1 embryos were transferred into 100 synchronized Hampshire ewes, of similar body size and body condition, to generate singleton pregnancies with minimal fetal and maternal phenotypic variation. Pregnant ewes were identified using abdominal ultrasound on gestational day (GD) 28. Ewes were weighed, isolated into individual concrete pens, and fed 100% of their nutritional requirement in a fully pelleted ration, based on body weight, for a one-week acclimation period. Ewes were then randomly divided into treatment groups, with 40 ewes fed a nutrient restricted diet [NR; 50% National Research Council (NRC) beginning on GD 35], and 13 fed 100% NRC. The NR ewes and 9 of the 100% ewes (control) were subject to a full placentomectomy surgeries. The remaining 4 ewes underwent a sham surgical procedure (sham). All ewes were weighed weekly, and diets adjusted according to body weight and requirements based on stage of gestation according to NRC guidelines.

### Surgical protocol

**Fig. 1:**
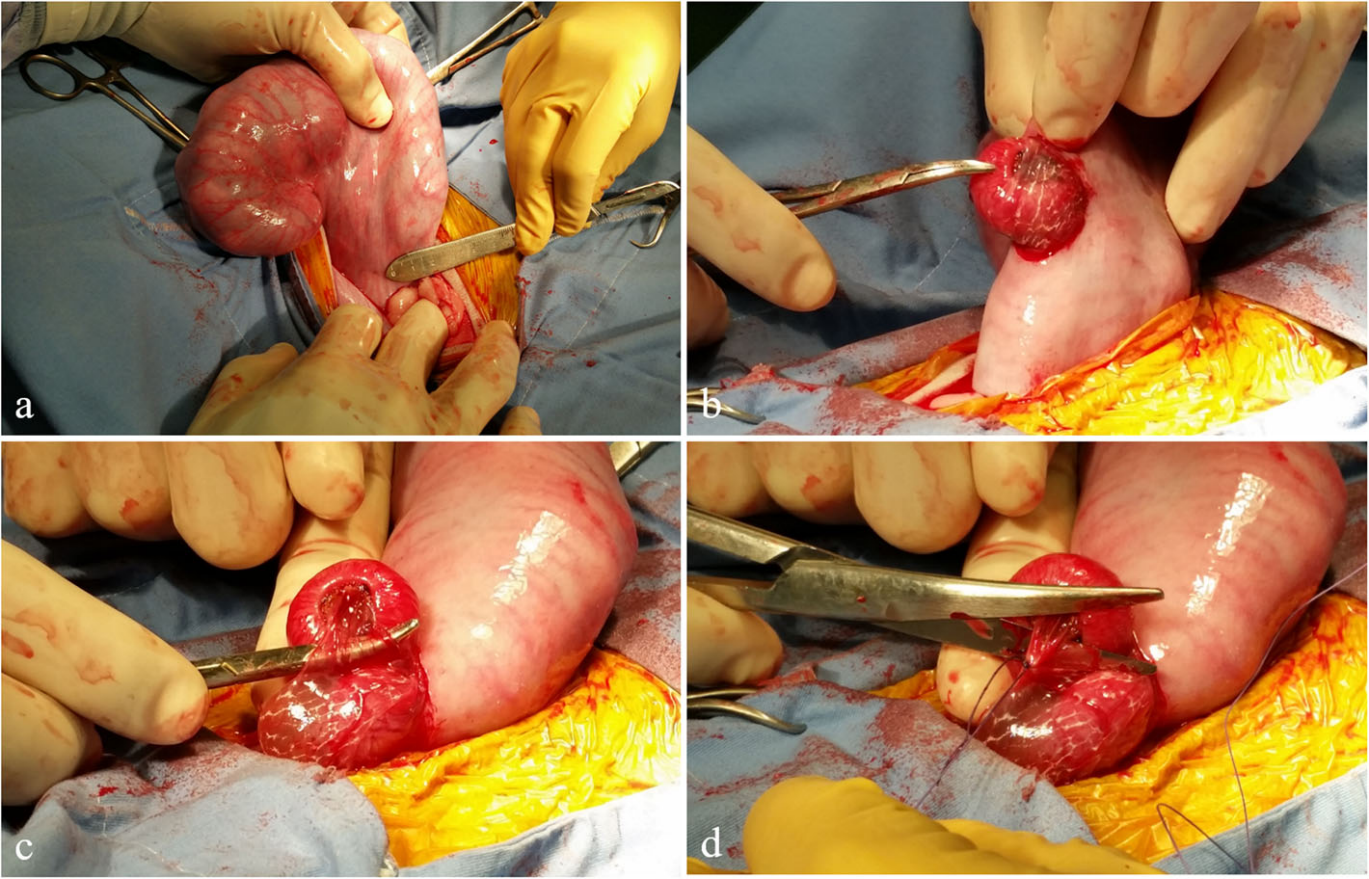
Entry into uterus and separation of fetal tissues from placentome. **a** The gravid uterine horn was exteriorized, and a placentome was identified just distal to the amniotic sac, and then (**b**) gently exteriorized through a scalpel incision. **c** The chorioallantois was isolated using hemostats and following ligation (**d**) the isolated membranes were transected

Ewes were fasted for 24 h prior to surgery. On GD 70, subjects were anesthetized using mask-delivered isoflurane until a surgical plane of anesthesia was achieved. Abdomens were clipped, shaved, and prepped using betadine and a 70% alcohol solution. Ioban drapes (3M Inc., Maplewood, MN, USA) were applied over the abdomen. A 10–15 cm ventral mid-line incision was made into the abdomen. Uterine horns were palpated, and the distal end of the gravid uterine horn was exteriorized (Fig. 1a) for aspiration of allantoic fluid. Fluid was collected using a 20G needle and a 5 cc syringe. Next, a placentome on the antimesometrial portion of the uterus was selected, near the conceptus, but distal to the edge of the amniotic sac. Identification of placentome type was not attempted prior to removal, but an effort was made to select one of representative size. An incision was made into the uterine wall at the proximal end of the placentome using a #10 scalpel blade.

**Fig. 2:**
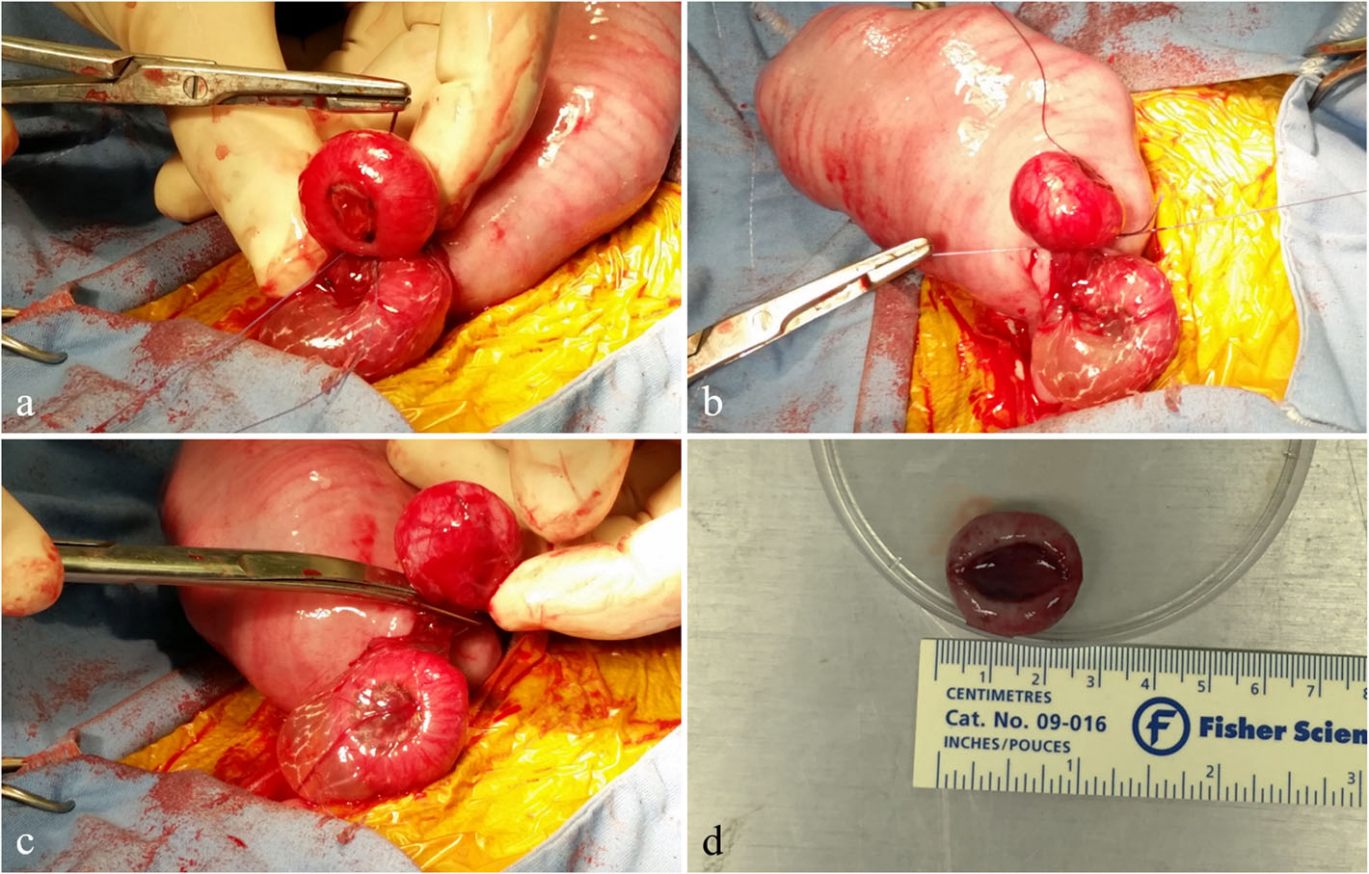
Separation of the maternal tissues from the placentome. **a** A modified transfixational suture was placed on the placentome stalk by passing a doubled length of suture through the stalk (**b**) and tying a ligature on both sides of the stalk. **c** The stalk of the placentome was transected and the stump was observed for bleeding. **d** Excised gestational d 70 placentome

In placentomectomy subjects, the remaining interplacentomal endometrial tissue was manually separated and the placentome was exteriorized (Fig. 1b). Curved mosquito hemostats were gently manipulated to pass beneath the chorioallantois (Fig. 1c), containing the vasculature to the selected placentome. A length of 2-0 vicryl was placed into the hemostats and pulled through to ligate around the blood vessels in the chorioallantois. Curved blunt-blunts were used to transect the tissue between the placentome and the ligature (Fig. 1d). A 2-cm tapered needle, threaded with one folded strand of 2-0 vicryl, was then passed through the placentome stalk (Fig. 2a). The threaded end of the suture was cut, resulting in 2 strands of suture passing through the stalk. Strands were used to ligate both sides of the stalk (Fig. 2b). The stalk was then transected between the ligatures and the placentome, and the preparation observed to ensure that there was no bleeding (Fig. 2c).

Four sham-operated ewes were subjected to an identical surgical protocol which concluded with the initial incision through the uterine wall. The placentomes were not exteriorized or removed, and the uterine incisions were closed as described.

**Fig. 3:**
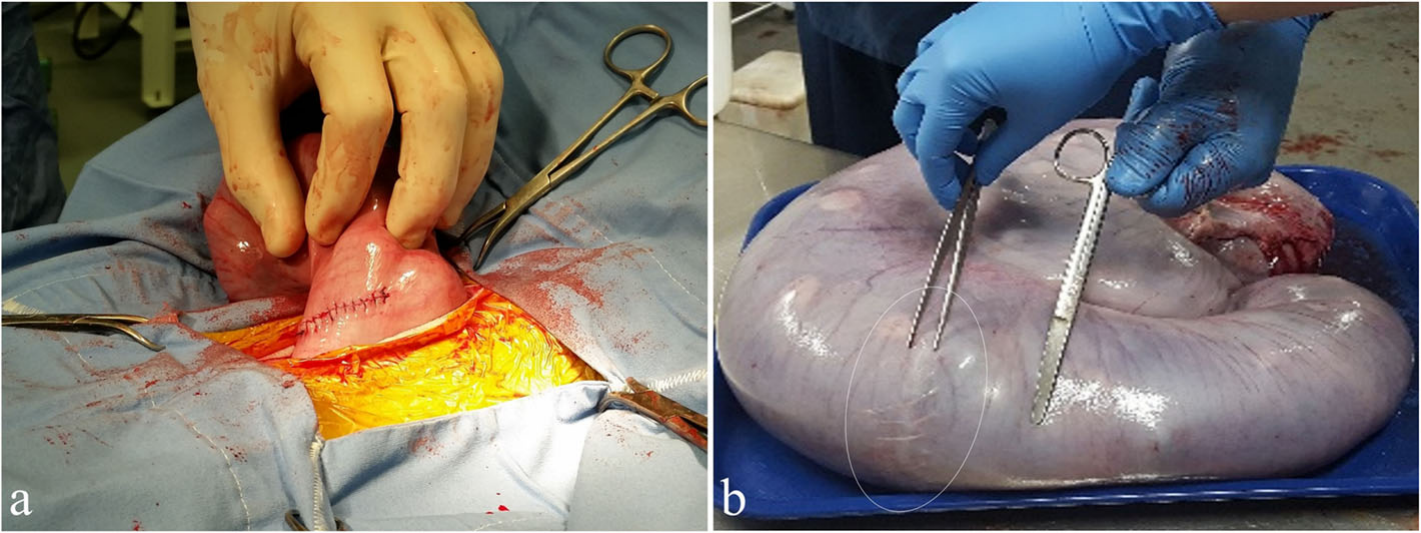
Operative incision healing. **a** The uterine wall was closed with a simple continuous pattern. **b** Healing of uterine wall incision at the time of necropsy. Forceps are pointing at the incision site, which is also circled and the blunt-blunts indicate the distal edge of the amniotic sac

In all ewes (placentomectomy and sham animals), the uterine incision was closed with 2-0 vicryl in a simple continuous pattern incorporating the submucosa, myometrium, and perimetrium (Fig. 3a). Warm 5% glycerol-saline was used to cleanse the uterine surface before the uterus was replaced into the abdominal cavity. The linea alba was closed using catgut in a Ford-Interlocking suture pattern. Subcutaneous fat was closed with catgut in a simple continuous pattern, and wound clips were used to close the skin incision. Ewes were held in a recovery area for 24 h before being returned to their original pens. Approximate average surgical time was 19 min from first incision to body wall closure. Anesthesia recordings were made every 5 min during the procedure, with an approximate cut and close time marked, and this data was extrapolated for an average. Excised placentomes (Fig. 2d) were weighed, and processed according to desired study protocols.

### Necropsy

Ewes were necropsied on GD 135. Surgical incisions (Fig. 3b) were evaluated at the time of necropsy, and additional fetal and maternal tissue samples were collected. Removed placentomes were counted and collectively weighed. Select central A-type placentomes were processed as desired for study purposes.

### Statistical analysis

Fetal and placentome weights and counts were compared between sham, control, and NR groups by analysis of variance using IBM SPSS Statistics for Windows, version 26 (IBM Corp., Armonk, NY, USA. All results are represented as mean ± standard error of the mean. A *P*-value of < 0.05 was considered significant.

## Results

### Wellness and measured outcomes were not different between control and sham ewes

**Fig. 4:**
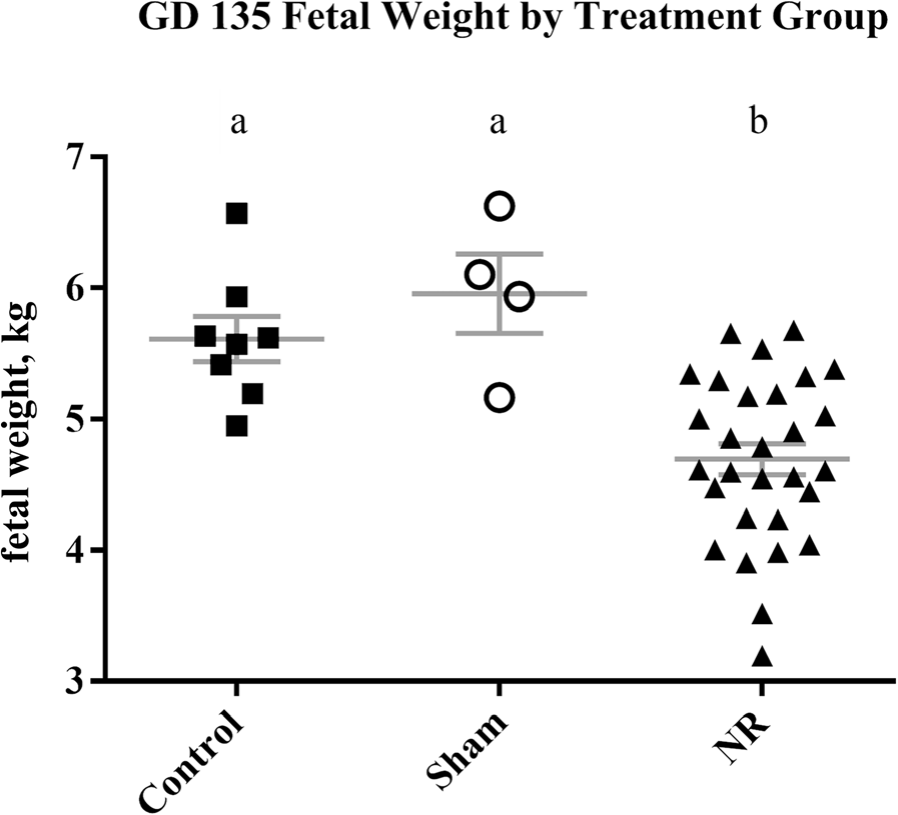
Fetal weights were not different between control ewes and sham ewes (*P* > 0.05). Fetal weights for nutrient restricted ewes were lower than for control (*P* < 0.05). GD is gestational day

There was no difference (*P* = 0.31) in fetal weights between control ewes and sham-operated ewes (Fig. 4). No complications from the procedure were observed in well-fed ewes. One control ewe produced a fetus with skeletal deformities, and was removed from the study. From the time that pregnancies were confirmed on GD 28, all ewes in the control and sham-operated groups maintained their pregnancies until the time of necropsy. Placentome numbers, weights, amniotic and allantoic fluid volumes, and maternal weight gain did not differ between control and sham ewes (*P* > 0.24; Table 1). While surgical incisions were made just beyond the edge of the amnion, at the time of necropsy the amnion had grown beyond the incision site in all subjects. Surgical time from incision to closure averaged 19 min. Evaluation of all surgical incisions showed complete healing. Regional placentomes were similar in appearance, size, and shape to those located distal to the incision.

**Table 1:** Control and nutrient restricted ewe comparative values

Category^c^	Control	Sham	50% NRC^d^
Ewes pregnant GD 28	9	4	41
Ewes necropsied GD 135	8	4	29
Avg fetal weight, kg	5.61 ± 0.17^a^	5.96 ± 0.30^a^	4.69 ± 0.12^b^
Crown-rump length, cm	64.12 ± 0.87^a^	65 ± 1.24^a^	60.27 ± 0.59^b^
Ponderal index	2.12 ± 0.05	2.16 ± 0.03	2.14 ± 0.04
Ewe weight change, kg	14.25 ± 2.20^a^	11.25 ± 2.69^a^	−32.41 ± 1.27^b^
Single placentome weight - GD 70, g	12.04 ± 1.22		10.57 ± 0.61
Placentome mass weight - GD 135, g	546.20 ± 43.32^a^	635.04 ± 50.25^a^	440.35 ± 21.89^b^
Placentome number - GD 135	80.87 ± 6.06	87.00 ± 5.44	78.10 ± 3.01
Placental efficiency	10.63 ± 0.67	9.46 ± 0.43	11.11 ± 0.38
Amniotic fluid volume, mL	651.88 ± 69.69	640.50 ± 39.84	778.52 ± 84.20
Allantoic fluid volume, mL	545.00 ± 115.12	702.00 ± 222.32	882.59 ± 123.34

^a,b^ Mean values in the same row not sharing the same superscript letters are significantly different (*P* < 0.05)

^c^GD is Gestational Day

^d^NRC is National Research Council nutritional requirement

### Nutrient restriction predictably impacted the treatment population

One nutrient restricted ewe aborted 3 d post-operatively. It was noted at the time of surgery that she had little amniotic fluid, minimal vasculature, a small fetus, and was likely losing her pregnancy. Respiratory issues were noted in one ewe and she was treated with antibiotics, but expired 4 days post-operatively. Nine additional nutrient-restricted ewes died or were euthanized in late gestation for reasons unrelated to the surgery. Fetal weights and crown-rump lengths for nutrient-restricted ewes were lower than those for fetuses from control ewes (4.69 ± 0.12 kg vs. 5.61 ± 0.17 kg; 60.27 ± 0.59 cm vs. 64.12 ± 0.88 cm; *P* < 0.05; Fig. 4), but ponderal indexes were not [PI = weight(g) × 100/height (cm)^3^; 2.14 ± 0.04 vs. 2.12 ± 0.05]. Weights of single placentomes were not different for ewes in the control (12.04 ± 1.22 g) and nutrient restricted (10.57 ± 0.61 g) groups (*P* > 0.3). Total placentome weight determined at necropsy was lower in NR ewes (546.20 ± 43.32 g control; 440.35 ± 21.89 g NR; *P* < 0.05; Table 1). Placentome numbers did not vary between treatment groups (80.87 ± 6.06 control; 78.10 ± 3.01 NR; *P* > 0.30), and were within the described range of 20–150 placentomes per ewe [6]. Placental efficiency [fetal weight (g)/placental weight (g)] was not different between treatment groups (10.63 ± 0.67 control; 11.11 ± 0.38 NR; *P* = 0.54). Nutrient restricted ewes lost a significant amount of weight compared to control ewes (14.25 ± 2.20 kg control; − 32.41 ± 1.27 kg NR; *P* < 0.01). At necropsy, amniotic and allantoic fluid volumes and colors were within the expected range from prior experiments, and did not differ between control, sham, or NR ewes (*P* > 0.16).

## Discussion

Collection of sufficient placental tissues for comprehensive analyses of both histological and gene expression changes in the early stages of maternal insult without compromising pregnancy has not been described. The development of such a novel technique is a critical step in developing the necessary tools to effectively investigate developmental origins of health and disease. Nutrient restricted ewe fetuses displayed the expected spectral variations in fetal development, where some ewes produced small for gestational age fetuses, and some produced fetuses closer to the weights observed for control and sham-operated ewes [9]. The phenomenon of spectral variation in fetal growth rates in response to maternal nutrient restriction has also been observed in cattle [12]. The surgical procedure allowed collection of placentomes to demonstrate differences in gene expression associated with size of the fetus, which is now being investigated. As removal of an entire placentome was possible, it is reasonable to believe that any research method previously validated for placentome analysis could be applied to excised placentomes as readily as a placentome collected during a GD 70 necropsy [13].

The use of a modified transfixational ligature for stalk removal ensured hemostasis at the site of removal of the placentome. The selection of a central and presumably highly functional placentome for removal was confirmed by their weight in comparison to the weight of placentomes at necropsy, as well as their location relative to the border of the amnion at the time of necropsy. We did not attempt to identify placentome type prior to removal as most placentomes should be concave or A-type on GD 70. Morphology is known to change later in gestation, but is primarily type A for the first two-thirds [6, 14]. The placentomectomy had no identifiable negative impact on fetal development in pregnant ewes when performed on GD 70. It is expected that NR itself might have altered placental efficiency, however the values were unchanged between NR, control, and sham ewes, indicating that the procedure itself did not cause drastic changes to efficiency. The procedure may have hastened progression of the disease in the nutrient restricted ewe which died 4 days post-operatively, as well as the abortion in the ewe which lost her pregnancy 3 days post-operatively. However, the placentomectomy itself is unlikely to have caused either event.

Placentome formation in the ewe occurs within the first two-thirds of pregnancy, with growth of the placenta plateauing near GD 90. By GD 70, the placenta has essentially achieved its full weight and completed its rapid growth phase. At this time, vascular development in the caruncular and cotyledonary tissues are also entering an exponential growth phase that continues throughout the last half of pregnancy [15]. Our selection of an essential time point for placentome sampling focused on the juncture of these significant growth stages. Concerns regarding stress and uterine manipulation were also factored into the choice of gestational stage for performing the placentomectomies. Placental production of progesterone is sufficient for maintenance of pregnancy by GD 60 and compliments production of progesterone by the corpora lutea [16]. Prior to GD 60, concentrations of progesterone are protective against uterine production of luteolytic prostaglandin F2alpha (PGF_2a_), but manual manipulation of the uterus may cause sufficient release of PGF_2a_ to trigger luteolysis and subsequent abortion. Minimal handling of the uterus during the placentomectomy procedure is recommended at any gestational age, especially when attempting a placentomectomy prior to GD 60.

There are potential concerns when performing this procedure. The above-mentioned risks of abortion due to uterine handling or maternal stress would compromise the potential of the longitudinal research technique to provide useful data on gene expression critical to subsequent fetal development. Failure to maintain hemostasis at the placentomectomy site, infection, anesthesia risks, and compromise to the amniotic and allantoic sacs are all considered risks of uterine surgery during gestation. Historically, carunclectomies have been performed prior to mating in the ewe model to produce IUGR fetuses. In those studies, a large percentage of caruncles were removed, which proved to be growth-restrictive in half of the ewes [1, 17, 18]. Sheep are considered polycotyledonary, having 20–150 smaller placentomes which occupying most, but not all available uterine caruncle sites [6]. As shown by the results of the present study, the removal of one placentome during gestation did not impact fetal size, uterine manipulation did not trigger abortion at GD 70, and hemostasis at the surgical site was sufficient to maintain a healthy pregnancy.

An alternative approach to further mitigate some of the mentioned risks would be a placentome biopsy instead of a placentomectomy. Due to the interdigitating and complex nature of placentomes, a concern would then be raised regarding the representative tissue in potential biopsy sections. Hemostasis at the biopsy site would also need to be considered. In species where the uterus is not accessed as readily as in ewes, a placentome biopsy may be an alternative but suboptimal research approach at least regarding the quantity and quality of tissue collected.

The ewe is a valuable established gestational research model [2, 7, 8]While some aspects of placentation and gestation are different from those in women, the low cost and ease of research with the ovine model have increased its appeal. Research areas span from fetoscopy studies to placentation and fetal programming studies, as evidenced in our NR treatment group. While the rodent or lagomorph models offer a seemingly more direct comparison to the hemochorial placenta of the human, the extended gestation period (21 d in the mouse, 31 d in the rabbit, 147 d in the ewe) in the ovine model provides for a better understanding of long-term developmental impacts on gestation. Similarly, the organogenesis timeline is a closer approximation for human development in the ewe than rodent or lagomorph models [19]. Placentomes have the added value of interdigitating maternal and fetal tissue sections that allows for growth and gene expression in these areas to be evaluated separately [20], although laser capture dissection or other approaches to single cell transcriptomics may be necessary to isolate tissue types due to the microscopic interdigitation and fusion, especially if performed later in gestation [21]. Comparisons of separate tissue types would not be possible in the more invasive tissues of the human or rodent placental disk. The availability of a procedure to sample placentomes while maintaining pregnancy, adds greatly to the worth of the sheep model in human and agricultural studies.

## Conclusions

The value of this technique can be most greatly realized when studying diseases that give a spectral phenotype. Placental changes can be used to predict and/or understand developmental challenges and ill-thrift in agricultural and human health research settings. Ruminant animals are the only ones that lend themselves to sampling of this nature. Given the low cost and effort involved in housing and feeding sheep, and their shorter gestation, ewes were the ideal animal in which to explore the technique for and value of placentomectomies. With a protocol and efficacy now established, the pregnant ewe stands to serve as an invaluable research model truly linking the histological and molecular signature of the placenta with subsequent penetrance of health or disease in adulthood of the same individual. Following comparisons of the tissues acquired through this pilot study, proof of the benefits of this sampling method to research will be irrefutable.

## Data Availability

The datasets used and/or analyzed during the current study are available from the corresponding author on reasonable request.

## Abbreviations

GD: Gestational day
IUGR: Intrauterine growth restriction
NRC: National Research Council
PGF_2a_: Prostaglandin F2alpha
